# Familial SCN1A‐Related Epilepsy in Palestinian Siblings: Challenges of Genetic Testing in Resource‐Limited Settings: A Case Report

**DOI:** 10.1002/ccr3.72926

**Published:** 2026-06-15

**Authors:** Anwar Abu Hetta, Raghad S. Razem, Shams Shahateet, Bushra Kh. Pujee, Amal M. Shawabka, Bara M. AbuIrayyeh

**Affiliations:** ^1^ Faculty of Medicine Palestine Polytechnic University Hebron Palestine; ^2^ Hebron Governmental Hospital Hebron Palestine

**Keywords:** autosomal dominant epilepsy, case report, Dravet syndrome, GEFS+, resource‐limited settings, SCN1A mutation, SCN1A‐related epilepsy

## Abstract

SCN1A‐related epilepsy may present in siblings with febrile and afebrile seizures. In resource‐limited settings, diagnosis relies heavily on clinical recognition and established criteria. Early identification and avoidance of sodium channel blockers are critical, while genetic testing, when available, supports diagnosis and family counseling.

## 1 Introduction

Dravet syndrome (DS), also known as severe myoclonic epilepsy of infancy, is a rare developmental and epileptic encephalopathy, with an estimated incidence of approximately 1 in 15,000–22,000 live births, marked by prolonged, recurrent seizures that are frequently precipitated by fever, vaccination, or intercurrent illness and often lead to progressive neurodevelopmental decline [1, 2].

The condition is most commonly linked to heterozygous pathogenic variants in the SCN1A gene, which encodes the voltage‐gated sodium channel Na(V)1.1 [3]. While most cases (approximately 90%) are caused by de novo variants [4], a smaller proportion (approximately 5%–10%) are inherited in an autosomal dominant pattern, with variable clinical expression and incomplete penetrance [5]. Symptoms typically appear in the first year of life in previously normally developing infants, progressing to multiple seizure types (including prolonged febrile seizures, myoclonic seizures, atypical absences, focal seizures, and generalized tonic–clonic seizures), pharmacoresistant epilepsy, cognitive slowing, motor impairment, and an elevated risk of sudden unexpected death in epilepsy (SUDEP) [6, 7].

Diagnosis of DS rests on a characteristic clinical presentation; genetic testing is supportive but neither required nor sufficient, as approximately 15%–20% of clinically diagnosed DS patients have no identifiable SCN1A variant, and SCN1A pathogenic variants can be associated with a spectrum of phenotypes beyond DS [6]. Confirmatory genetic testing, such as whole exome sequencing (WES), can identify pathogenic SCN1A variants [6]. Treatment is often complex, involving multiple agents such as valproate, clobazam, stiripentol, cannabidiol, or fenfluramine, while avoiding sodium channel blockers (e.g., carbamazepine, phenytoin, and lamotrigine) that can aggravate seizures. Established nonharmacological options include the ketogenic diet, which has strong clinical evidence in DS. Emerging experimental approaches include gene‐targeted therapies [6]. In resource‐limited contexts, restricted access to genetic testing limits confirmation of underlying genetic etiology, complicating counseling and long‐term care planning [8]. We describe two siblings from a non‐consanguineous Palestinian family who developed recurrent seizures following febrile episodes. One sibling received a confirmed SCN1A pathogenic variant identification through whole exome sequencing (WES), meeting clinical criteria for Dravet syndrome, while the other was diagnosed clinically as presenting within the genetic epilepsy with febrile seizures plus (GEFS+) spectrum.

## 2 Case Presentation

### 2.1 Case History and Examination

#### 2.1.1 Patient 1

The first patient is a 4 year‐old Palestinian girl, born at 38 weeks of gestation via cesarean section, with a birth weight of 3.3 kg. Her perinatal course was unremarkable. At 4 months of age, she was hospitalized following a focal motor seizure involving the right side of the body after a febrile episode that occurred following routine immunization. At 6 months of age, she experienced another generalized tonic–clonic seizure associated with fever, also after immunization. During both episodes, she received intravenous diazepam and antipyretics.

Thereafter, she developed increasingly frequent generalized tonic–clonic seizures occurring both with and without fever, typically lasting 3–4 min and followed by a brief postictal phase. At 14 months of age, she was admitted with status epilepticus characterized by repetitive generalized tonic–clonic seizures. During hospitalization, she subsequently developed episodes of eye deviation, mouth twitching, unresponsiveness, oxygen desaturation, and focal right‐hand seizures.

At 2.5 years of age, she experienced another episode of status epilepticus. At her most recent follow‐up (4 years and 2 months of age), she was meeting age‐appropriate developmental milestones, including speaking in full sentences, engaging in imaginative play, and demonstrating age‐appropriate gross and fine motor skills. Physical examination revealed no dysmorphic features or organomegaly, and neurological examination was normal.

#### 2.1.2 Patient 2

The second patient is a 9‐month‐old Palestinian boy, the younger sibling of Patient 1. He was born at 38 + 4 weeks of gestation via cesarean section because of previous uterine scars and weighed 3.7 kg at birth. His perinatal course was notable for maternal gestational diabetes mellitus.

At 7 months of age, he experienced a generalized tonic–clonic seizure during a febrile illness, presenting with sudden shoulder flexion and spasm‐like movements lasting 3–4 min, followed by a brief postictal period and oxygen desaturation. He was admitted to hospital and treated with intravenous diazepam, antibiotics, and antipyretics. 1 month later, he experienced a similar febrile seizure episode.

At his most recent follow‐up (9 months of age), he was achieving age‐appropriate developmental milestones, including independent sitting, babbling, and responding to his name. Physical examination showed no dysmorphic features or organomegaly. Neurological, ophthalmological, and hearing examinations were normal.

### 2.2 Family History

The patients' father, currently 23 years old, has a history of poorly controlled seizures beginning in infancy and reportedly has mild intellectual disability without physical impairment. Genetic testing was not performed because of financial constraints. The maternal uncle also reportedly has a history of seizures. The parents are non‐consanguineous.

### 2.3 Differential Diagnosis

For Patient 1, the differential diagnosis included recurrent febrile seizures, central nervous system infection, metabolic causes of seizures, genetic epilepsy with febrile seizures plus (GEFS+), and Dravet syndrome. The early onset of seizures, occurrence of both febrile and afebrile seizures, recurrent status epilepticus, and worsening following sodium channel blocker therapy favored a diagnosis within the SCN1A‐related epilepsy spectrum, specifically Dravet syndrome.

For Patient 2, the differential diagnosis included recurrent febrile seizures, GEFS+, and an evolving SCN1A‐related epilepsy syndrome. At the time of assessment, he did not fulfill clinical criteria for Dravet syndrome and had not yet met diagnostic criteria for epilepsy.

### 2.4 Investigations

#### 2.4.1 Patient 1

Initial and follow‐up electroencephalograms showed no epileptiform activity. During hospitalization for status epilepticus, blood gas analysis, serum electrolytes, blood glucose, liver function tests, and complete blood count were within normal limits. Serum ammonia was elevated at 67 μmol/L (reference range 15–45 μmol/L). Cerebrospinal fluid analysis was unremarkable.

Brain magnetic resonance imaging performed at 2 years of age showed no abnormalities. Ophthalmological and hearing assessments were normal.

Whole exome sequencing performed as a proband‐only analysis identified a heterozygous pathogenic SCN1A variant, c.302G >A (p.Arg101Gln). Together with her clinical features, these findings supported a diagnosis of Dravet syndrome.

#### 2.4.2 Patient 2

Electroencephalography revealed no epileptiform abnormalities. Whole exome sequencing was not performed because of financial constraints.

### 2.5 Treatment

#### 2.5.1 Patient 1

During the initial seizure episodes, the patient received intravenous diazepam and antipyretics. During admission for status epilepticus, she received two doses of intravenous diazepam followed by a loading dose of phenytoin. After subsequent seizure recurrence, additional intravenous diazepam was administered. Carbamazepine was introduced but was associated with paradoxical seizure worsening. Seizure control was subsequently achieved with intravenous sodium valproate, levetiracetam, and phenobarbital.

Intravenous acyclovir was initiated while preparing for lumbar puncture and was continued until cerebrospinal fluid analysis excluded central nervous system infection. Following stabilization, she was discharged on oral sodium valproate and continued neurological follow‐up.

#### 2.5.2 Patient 2

During both febrile seizure episodes, the patient received supportive treatment including antipyretics and intravenous diazepam. Long‐term antiseizure medication was not initiated because he had not yet met clinical criteria for epilepsy. Ongoing management consists of clinical surveillance, seizure education, and regular pediatric neurology follow‐up.

### 2.6 Family History

The patients' father, now 23 years old, has a longstanding history of poorly controlled seizures with onset in infancy and a reported mild intellectual disability; he has no physical impairments. Whole exome sequencing (WES) was not performed in him due to financial constraints. Additionally, the maternal uncle has a reported history of seizures. There is no other significant family history. The parents are non‐consanguineous.

## 3 Discussion

Dravet syndrome, first described by Dravet in 1978 [9], is a severe epileptic encephalopathy characterized by onset of multiple seizure types—most commonly generalized tonic, clonic, or tonic–clonic—within the first year of life in previously healthy children. Seizures are frequently triggered by fever, minor infections, or immunization and are often refractory to standard antiseizure therapy [10]. Over time, affected individuals may develop additional seizure types including absence, myoclonic, and focal seizures. Pathogenic variants in the SCN1A gene, encoding the α‐subunit of the neuronal voltage‐gated sodium channel, are identified in approximately 80% of cases [11].

It is important to emphasize that a Dravet syndrome diagnosis is clinical: an SCN1A pathogenic variant is neither required (approximately 15%–20% of clinically diagnosed DS patients have no identified variant) nor sufficient (SCN1A variants are associated with a spectrum of phenotypes including GEFS+ and other epilepsies). Conversely, the clinical diagnosis can and should be made based on fulfillment of established criteria even in the absence of genetic testing.

Clinical diagnostic criteria for Dravet syndrome, derived from population‐based studies, require fulfillment of at least 4 of the following 5 criteria: (1) normal neurodevelopment before seizure onset; (2) two or more seizures before age 12 months; (3) myoclonic, hemiclonic, or generalized tonic–clonic seizures; (4) two or more seizures lasting longer than 10 min; and (5) refractory seizures after age 2 years [12].

Patient 1 fulfills ≥ 4 of these 5 criteria: (1) normal early neurodevelopment confirmed; (2) two febrile seizures before age 12 months (at 4 and 6 months); (3) generalized tonic–clonic and focal motor seizures; (4) multiple episodes of status epilepticus lasting > 10 min; and (5) refractory seizures persisting past age 2 years. She therefore fulfills clinical diagnostic criteria for Dravet syndrome, supported by an identified pathogenic SCN1A variant (c.302G >A; p.Arg101Gln).

Patient 2, at 9 months of age, currently meets only 2 of 5 criteria (normal early development; 2 febrile seizures before age 12 months). He does not yet fulfill clinical criteria for Dravet syndrome. His current presentation is most consistent with genetic epilepsy with febrile seizures plus (GEFS+), which represents the milder end of the SCN1A phenotypic spectrum. Close follow‐up is warranted, as the clinical picture may evolve. The strong family history (affected sibling with confirmed pathogenic SCN1A variant, father with infantile‐onset epilepsy) elevates his a priori risk of an SCN1A‐related epilepsy considerably.

The diagnosis in Patient 1 was supported through genetic testing, which revealed a heterozygous pathogenic missense variant, SCN1A c.302G >A p. (Arg101Gln). This variant results in substitution of arginine with glutamine at position 101, previously reported as disease‐causing for severe myoclonic epilepsy of infancy [13, 14]. It is classified as pathogenic (class 1) per ACMG recommendations and is listed in ClinVar (accession VCV000006824.6) with a pathogenic interpretation [15].

In the context of the father's history of early infantile seizures and the recurrence of epilepsy in multiple family members, these findings strongly support an autosomal dominant (AD) inheritance pattern, consistent with SCN1A‐related epilepsies, where affected individuals have a 50% risk of transmitting the pathogenic variant to offspring [16]. Although parental genetic testing was not performed due to financial constraints, and the WES was proband‐only, the clinical evidence makes paternal inheritance highly probable.

Familial and sibling cases of SCN1A‐related Dravet syndrome have been documented worldwide, as summarized in Table 1. Most reports involve two affected siblings with onset during infancy (5–12 months), presenting with febrile hemiclonic or generalized seizures evolving to multiple seizure types. Recurrence is frequently explained by parental mosaicism or, less commonly, biallelic variants in consanguineous families [17, 18, 19, 20, 21, 22, 23]. The p.Arg101Gln variant closely parallels our Patient 1 in both genotype and clinical features [13, 14], reinforcing its pathogenicity.

**TABLE 1:** Reported sibling cases of SCN1A‐related epilepsy and comparison with the present cases.

Author (Year)	No. of siblings	Age at onset	Seizure types	SCN1A variant	Inheritance pattern	EEG findings
Sharkia et al. [17]	2	5–8 months	Febrile hemiclonic → GTCs, myoclonic, absence	Novel pathogenic variant (details in source)	Parental mosaicism	Multifocal epileptiform discharges
Selmer et al. [18]	2	6–9 months	Febrile GTCs, myoclonic	Novel; parental mosaicism	Parental mosaicism	Generalized spike–wave
Tuncer & Yilmaz [19]	2	5–7 months	Febrile hemiclonic, GTCs	c.302G>A p. (Arg101Gln)	Autosomal dominant	Generalized spike–wave
Kimura et al. [20]	2 brothers	8–12 months	GTCs, myoclonic, focal	Missense; paternal inheritance	Autosomal dominant (paternal FS)	Generalized epileptiform discharges
Hernandez et al. [21]	2	6 months	Febrile GTCs → GEFS+ phenotype	Homozygous likely pathogenic variant	Biallelic (consanguineous)	Normal/mild changes
Xu et al. [23]	2	7–10 months	Febrile GTCs, myoclonic	Multiple; parental mosaicism	Parental mosaicism	Generalized spike–wave
Present cases (2025)	2 (F & M)	4–7 months	Febrile focal/GTCs, SE; Patient 2: febrile GTCs only	c.302G>A p. (Arg101Gln) in Patient 1; not tested in Patient 2	Likely AD (paternal)	Normal EEG in both

Abbreviations: AD, autosomal dominant; DS, Dravet syndrome; EEG, electroencephalogram; F, female; FS, febrile seizures; GEFS+, genetic epilepsy with febrile seizures plus; GTCs, generalized tonic–clonic seizures; M, male; mo, months; SCN1A, sodium channel neuronal type 1 alpha subunit gene; SE, status epilepticus; WES, whole exome sequencing.

In high‐resource settings, parental and sibling genetic testing would typically be performed to determine whether a variant is de novo or inherited, enabling accurate recurrence risk estimation and genetic counseling. In this Palestinian family, financial constraints prevented further testing. Notably, financial constraints also limited treatment options: stiripentol and fenfluramine, which are recommended adjunctive agents in DS management guidelines, were not available locally, and supervised ketogenic diet therapy could not be accessed. This highlights both clinical and socioeconomic disparities in low‐resource settings.

Despite incomplete genetic workup, we believe these families should still receive counseling about empiric recurrence risk. Given the father's phenotype and the confirmed pathogenic variant in Patient 1, the family should be counseled that subsequent offspring carry an estimated 50% recurrence risk, pending formal parental testing if and when it becomes accessible.

### 3.1 Treatment Considerations

Management in both patients illustrates the therapeutic challenges of Dravet syndrome. In Patient 1, both phenytoin (used during her initial status epilepticus episode, before DS was suspected) and carbamazepine (administered subsequently) are sodium channel blockers known to aggravate SCN1A‐related epilepsies [24]. The paradoxical worsening observed with carbamazepine served as a key diagnostic clue. This underscores why early clinical recognition of DS—even prior to genetic testing—is critical: avoidance of sodium channel blockers should be initiated as soon as DS is clinically suspected, without waiting for genetic confirmation.

Comparing our treatment approach to the 2022 international consensus guidelines for DS management [25], deviations from recommended practice included: (1) use of phenytoin for initial status epilepticus management (guideline‐preferred agents are benzodiazepines followed by valproate or levetiracetam); (2) inability to initiate clobazam as first‐line adjunctive therapy due to local availability; and (3) inability to access stiripentol, fenfluramine, or cannabidiol due to cost and unavailability in the local formulary. Transition to valproic acid provided partial benefit, with subsequent use of levetiracetam, clobazam where available, and topiramate reflecting the frequent requirement for multidrug regimens [25]. IV diazepam was used as rescue therapy in both siblings [25].

In Patient 2, who has had two provoked febrile seizures and does not yet meet clinical criteria for epilepsy, withholding daily antiseizure medications is clinically appropriate at this stage. The rationale for non‐initiation is the absence of an epilepsy diagnosis, not simply seizure infrequency—even patients with infrequent seizures who meet epilepsy criteria should be considered for treatment. Management focuses on acute rescue therapy (rescue benzodiazepines for prolonged seizures), fever management education, and close neurology follow‐up.

## 4 Conclusions

This report highlights several critical lessons. First, clinical recognition of Dravet syndrome using established diagnostic criteria is possible—and essential—even in the absence of genetic testing. Clinicians in resource‐limited settings should be familiar with the 5‐criterion clinical diagnostic framework. Second, sodium channel blockers (carbamazepine, phenytoin, lamotrigine) must be avoided in any patient with suspected SCN1A‐related epilepsy, as their use can precipitate or worsen seizures—the paradoxical worsening observed in Patient 1 was itself a diagnostic clue. Third, when a pathogenic SCN1A variant is identified in a proband, families should be counseled about the 50% recurrence risk for offspring even when parental testing is unavailable; phenotypic information alone can inform this counseling. Fourth, the inability to access optimal therapies and genetic services in low‐income settings represents a significant and addressable health equity gap. Advocacy for expanded access to both genetic diagnostics and guideline‐recommended treatments is urgently needed.

## Author Contributions

**Anwar Abu Hetta:** conceptualization, data curation, formal analysis, supervision, validation, writing – review and editing. **Raghad S. Razem:** conceptualization, data curation, formal analysis, investigation, writing – original draft. **Shams Shahateet:** conceptualization, data curation, formal analysis, investigation, methodology, writing – original draft. **Bushra Kh. Pujee:** conceptualization, data curation, formal analysis, investigation, methodology, writing – original draft. **Amal M. Shawabka:** conceptualization, data curation, investigation, methodology, writing – original draft. **Bara M. AbuIrayyeh:** conceptualization, data curation, formal analysis, investigation, methodology, writing – original draft.

## Funding

The authors have nothing to report.

## Ethics Statement

The authors have nothing to report.

## Consent

Written informed consent was obtained from the patients' parents/legal guardians for publication of this case report and accompanying clinical information.

## Conflicts of Interest

The authors declare no conflicts of interest.

## Data Availability

The data used to support the findings of this study are included in the article.
